# Improving polio vaccination during supplementary campaigns at areas of mass transit in India

**DOI:** 10.1186/1471-2458-10-243

**Published:** 2010-05-11

**Authors:** Naman K Shah, Ashok Talyan, Vibhour Jain, Sunil D Khaparde, Sunil Bahl, Yvan Hutin, Jay Wenger

**Affiliations:** 1 School of Medicine, University of North Carolina, Chapel Hill, USA; 2 National Polio Surveillance Project, New Delhi, India; 3 Ministry of Health and Family Welfare, Government of India, New Delhi, India; 4 Field Epidemiology Training Program, World Health Organization, New Delhi, India

## Abstract

**Background:**

In India, children who are traveling during mass immunization campaigns for polio represent a substantial component of the total target population. These children are not easily accessible to health workers and may thus not receive vaccine. Vaccination activities at mass transit sites (such as major intersections, bus depots and train stations), can increase the proportion of children vaccinated but the effectiveness of these activities, and factors associated with their success, have not been rigorously evaluated.

**Methods:**

We assessed data from polio vaccination activities in Jyotiba Phule Nagar district, Uttar Pradesh, India, conducted in June 2006. We used trends in the vaccination results from the June activities to plan the timing, locations, and human resource requirements for transit vaccination activities in two out of the seven blocks in the district for the July 2006 supplementary immunization activity (SIA). In July, similar data was collected and for the first time vaccination teams also recorded the proportion of children encountered each day who were vaccinated (a new monitoring system).

**Results:**

In June, out of the 360,937 total children vaccinated, 34,643 (9.6%) received vaccinations at mass transit sites. In the July SIA, after implementation of a number of changes based on the June monitoring data, 36,475 children were vaccinated at transit sites (a 5.3% increase). Transit site vaccinations in July increased in the two intervention blocks from 18,194 to 21,588 (18.7%) and decreased from 16,449 to 14,887 (9.5%) in the five other blocks. The new monitoring system showed the proportion of unvaccinated children at street intersection transit sites in the July campaign decreased from 24% (1,784/7,405) at the start of the campaign to 3% (143/5,057) by the end of the SIA, consistent with findings from the more labor-intensive post-vaccination coverage surveys routinely performed by the program.

**Conclusions:**

Analysis of vaccination data from transit sites can inform program management changes leading to improved outcomes in polio immunization campaigns. The number of vaccinated children encountered should be routinely recorded by transit teams and may provide a useful, inexpensive alternative mechanism to assess program coverage.

## Background

Since the launch of the global polio eradication program in 1988, the incidence of paralytic polio has decreased by more than 99% and three of six WHO regions have been certified as polio-free [1]. India reported 741 cases in 2009. This represented 59% of the total burden in the four remaining endemic countries (Afghanistan, India, Nigeria, and Pakistan) [2]. Continued transmission of wild poliovirus was identified in India in early 2010, and achieving polio eradication will require continued attention to high vaccination coverage among the under five population. During supplementary immunization activities (SIAs - or mass immunization campaigns) oral polio vaccine is offered to all children under five years of age, irrespective of previous vaccination status. Once vaccinated, children are marked with indelible ink on their left small finger. Vaccine administration begins on a Sunday at fixed sites ("booths", where parents are encouraged through mass media to bring their children for immunization) and is followed with several days of visits to all houses in the area by vaccination teams to vaccinate children who were not vaccinated during the booth day (house-to-house vaccination). However, children that are traveling with their families during the SIA might miss their vaccination during booth and house-to-house activities.

Common areas of mass transit in India include street intersections, bus stands, and railway stations. India introduced transit immunization teams in 2004 to vaccinate children who were traveling at these sites during the SIA as part of a broader strategy to reach underserved populations. The overall strategy resulted in an additional five million children being vaccinated that year [3]. In addition, during sub-national immunization days in the polio endemic states of Bihar and Uttar Pradesh, simultaneous SIAs are now conducted in other high risk areas of states including Delhi, Mumbai, and Kolkata to which many families from Bihar and Uttar Pradesh migrate. Data suggests the continued strengthening of transit vaccination strategy is crucial as India progresses towards eliminating poliovirus. An analysis of serotype 1 wild polio virus cases outside of Uttar Pradesh and Bihar during 2007-2009 found that more than 40% of cases were among mobile and migrant populations. Acute flaccid paralysis surveillance data shows the cases of acute flaccid paralysis among mobile and migrant populations have fewer previous polio vaccinations than cases from stable populations [unpublished data, National Polio Surveillance Project].

The polio eradication program collects and analyzes data to translate information into action at district, state, and national levels. For example, the analysis of surveillance and SIA monitoring data is used to determine the geographic extent of each SIA and the vaccination strategies used in each area. However, since the transit team strategy was introduced only recently, the program is just beginning to use data to evaluate operational aspects of the strategy. Our study evaluates the use of transit site vaccination data for effective local interventions.

We collected data from two mass vaccination activities during June and July 2006 at areas of mass transit in Jyotiba Phule (JP) Nagar district in the state of Uttar Pradesh to describe transit activities and determine whether the analysis of transit vaccination data could benefit decision-making. First, we reviewed transit vaccinations to estimate the proportion of total vaccinations achieved at transit sites and to examine the association between polio vaccine delivery and various factors (e.g., type of transit site, community composition of teams). Second, we used data on the number of children administered vaccine during the first activity in June to plan the timing, location, and human resource requirements of vaccination shifts for two of the seven blocks of the district during the second activity in July. Third, we compared the two intervention blocks with the five others in terms of number of children vaccinated during the July immunization activities to determine whether the use of the June data to inform SIA activities resulted in better performance.

## Methods

### Geographic area

JP Nagar district (2001 population: 1.49 million; population density: 607 persons/square kilometer) [4] in Western Uttar Pradesh (UP) is endemic for poliovirus transmission. The division of UP that includes JP Nagar district was the epicenter of the 2006 polio outbreak and wild poliovirus from this area spread to other areas [5]. The population is 60% Hindu and 39% Muslim, and 75% rural with an average household size of seven persons. Literacy proportions are 63% and 35% among males and females, respectively. It is divided in seven administrative blocks for the polio program (average population size: 213,000).

### Data sources and ethics

We used routine data collected by the districts, state governments, and the National Polio Surveillance Project. Aggregate measures are publicly available on the website of the latter http://www.npspindia.org. The specific data used in this work can be made available upon request. The study was conducted as an evaluation of an ongoing public health program and therefore IRB exempt.

### Review of the June 2006 data

We obtained original vaccination sheets recorded by individual transit site vaccination teams from each of the seven administrative blocks for the June 2006 SIA. We selected transit vaccination data from two of the blocks, Amroha Urban and Gajraula, for analysis, since they had the most transit sites. We entered information from individual vaccinator record sheets into a database including: block, team number, transit site, date, number of children vaccinated, type of site (bus stand, street intersection, or rail station), and presence of at least one Muslim vaccinator as a team member.

### Use of data for decision-making

Based on the June data we introduced interventions, centralized management and planning to improve vaccine delivery to children (i.e., to increase the number of children vaccinated). For the analysis of the June data, we excluded teams in which members were changed during the course of the SIA (which would result in a change of the team composition category) and we also excluded teams identified by supervisors as falsifying vaccination sheets during monitoring visits. We examined the number of children vaccinated at each transit site during the June SIA to plan the length of time of each vaccinator shift, human resource requirements, and activity timing for the subsequent July SIA. For example, transit sites with few children vaccinated relative to other sites in that block were either discontinued or the number of teams at that site was reduced. At transit sites where many children continued to be vaccinated throughout the course of the SIA, the number of teams was increased or multiple shifts of shorter durations were created so that vaccinators would not tire as quickly. In addition, a single manager was assigned to perform planning, training of vaccinators, and monitoring functions in the two intervention blocks (Amroha Urban and Gajraula) for the July SIA. This was in contrast to the usual procedure of having these activities performed by the existing block level health officials (who continued to perform these functions in the non-intervention blocks). We also compared vaccination teams with and without Muslim team members in terms of the number of children vaccinated per shift at different types of transit sites in the Amroha Urban and Gajraula blocks in the June SIA using a two-sided Wilcoxon rank-sum test. Efforts were made to ensure that all teams had at least one Muslim vaccinator for the July round.

After the end of the July SIA we collected data and entered it as previously described. In addition, we collected information on the proportion of children encountered at transit sites vaccinated during the SIA.

### Impact analysis

We compared June and July data in the intervention blocks (Amroha Urban and Gajraula) and the non-intervention blocks (Amroha Rural, Dhanura, Joya, Hasanpur, and Rehra blocks), in terms of the average number of children vaccinated per team-shift (one shift of work for a team of two vaccinators). We divided the total number of children vaccinated by the total team-shifts to correct for the effects of changes in human resources on the number of children administered vaccine.

We calculated the proportion of children vaccinated at transit sites by dividing the total transit vaccinations by the total number of vaccinations. We calculated the median number of vaccinations per team-shift at railway sites. We calculated the proportion of children unvaccinated by date and by the type of transit site on the last day of the July SIA vaccination activity. We identified sites where unvaccinated children were no longer encountered as the SIA progressed to determine where transit site teams could be reduced during the last days of "mop-up" vaccination activities at the end of the SIA.

## Results

### June 2006 SIA

**Figure 1:**
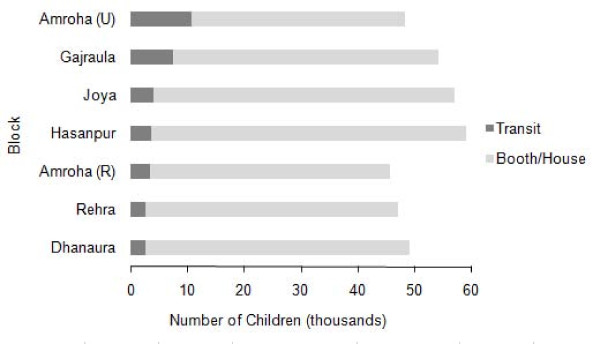
**Number of children vaccinated at transit sites and vaccinated at booths or homes by block, Jyotiba Phule Nagar district, Uttar Pradesh, India, June 2006**.

In JP Nagar district, polio vaccination teams vaccinated 34,643 children at mass transit sites and this accounted for 9.6% of the total 360,937 children vaccinated. The proportion of vaccinations performed at transit sites ranged from 5% (2,671/49,204) in Dhanaura block to 22% (10,696/48,271) in Amroha Urban block (Figure 1). A total of 806 team-shifts of vaccinators administered OPV to 16,449 children at transit sites in Amroha Rural, Dhanura, Hasanpur, Joya, and Rehra blocks. In Amroha Urban and Gajraula blocks, 882 team-shifts of vaccinators administered vaccine to 18,194 children at transit sites. Both sets of blocks vaccinated a similar number of children per team-shift (20.4 vs. 20.6 respectively). In Amroha Urban teams at railway stations with at least one Muslim member vaccinated on average 13 more children per shift than others (z = 1.98, P = 0.048)(Table 1). The average number of children vaccinated also varied with the team composition at bus stands or street intersections but the difference was not significant (z = 1.136, P = 0.256 and z = 0.586, P = 0.558 respectively). None of the transit teams in Gajraula had any Muslim team members.

**Table 1:** Median number of vaccinations per shift according to team composition, Amroha block, Jyotiba Phule Nagar district, Uttar Pradesh, India, June 2006.

Type of Site	Median number vaccinated by team		Δ
		
	Without any Muslim member	At least one Muslim member	
Rail station	41	54	13*
Street intersection	50	42	-8
Bus stand	65	59	-6

*p < 0.05

### Data driven program changes

On the basis of the analysis of the June 2006 data, a number of changes were implemented in Amroha Urban and Gajraula for the July SIA by a single district level manager. More transit sites, additional transit teams, and shorter vaccination shifts were employed in areas where teams continued to deliver a high number of vaccinations until the end of the SIA in June. Conversely, the number of sites and human resources were decreased in areas where vaccination activity was low (or declined rapidly in the previous SIA). Previously, the addition of sites, allocation of teams, and timings of the shifts in these two blocks were determined on an ad hoc basis by the block level manager. Non-intervention blocks continued to plan transit operations in this manner. Muslim vaccinators were added to teams at railway sites that had no Muslim vaccinators so that all transit teams at railway sites included at least one Muslim team member in the July SIA. Finally, for future data driven decisions, additional monitoring data was sought during the campaign. Transit vaccination teams in Gajraula, in addition to routine data collection, were instructed to record the number of children encountered who had already been vaccinated during the July SIA to estimate the proportion of unvaccinated children remaining each day.

### July 2006 SIA

Overall, polio vaccination at mass transit sites reached 36,475 children and accounted for 9.9% of the total 368,071 children vaccinated. Thus, an additional 5.3% (1,832/34,643) children received vaccinations at JP Nagar mass transit areas in July compared with June.

In the non-intervention blocks of Amroha Rural, Dhanura, Hasanpur, Joya, and Rehra, 847 team-shifts of vaccinators reached 14,887 children at transit sites. The number of doses administered at transit vaccinations sites declined by 9.5% (1,562/16,449) compared to June. The number of children vaccinated per team-shift also decreased from 20.4 to 17.4 compared to the June SIA.

**Figure 2:**
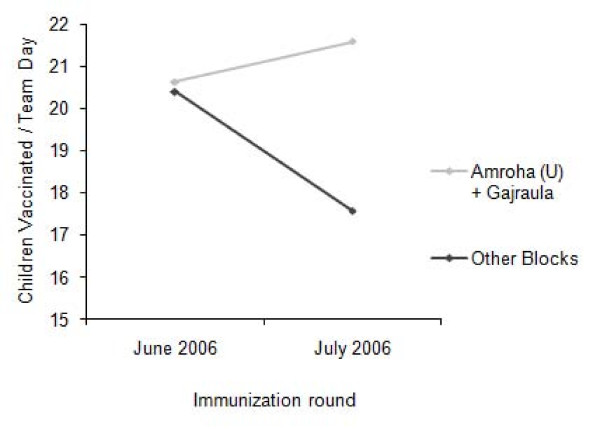
**Change in children vaccinated per team-shift in Amroha Urban and Gajraula (intervention blocks) versus others, Jyotiba Phule Nagar district, Uttar Pradesh, India, June-July 2006**.

In the intervention blocks Amroha Urban and Gajraula, 1,000 team-shifts of vaccinators covered 21,588 children at transit sites. Transit vaccinations increased by 18.7% (3,394/18,194) compared to June. The number of children vaccinated per team-shift increased from 20.6 to 21.6 (Figure 2) compared to June. In Amroha, where all railway teams now included at least one Muslim member, a median of 57 children were vaccinated per shift compared to 54 children per shift in June.

**Figure 3:**
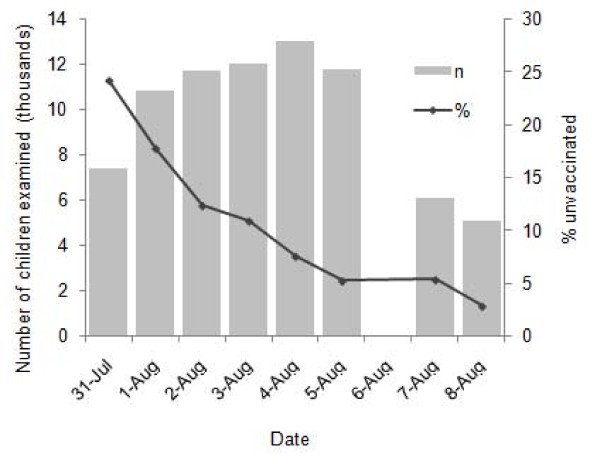
**Proportion of unvaccinated children and total number examined for vaccination status over the course of the SIA, Gajraula block, Jyotiba Phule Nagar district, Uttar Pradesh, India, July 2006**.

In Gajraula, the new monitoring data collected by transit teams examined the vaccination status of an average of 9,700 children per day. The number of unvaccinated children progressively declined over the course of the July immunization activities (Figure 3). The number of children examined declined after last day of the campaign due to a decrease in the number of transit teams involved by the end of the SIA. The program achieved 97% (4,914/5,057) vaccination coverage amongst children at mass transit sites by the end of the July SIA. On the final day of vaccination activities, children seen at railway sites and bus stands were at least two times more likely to be unvaccinated than those seen at street intersections (Table 2).

**Table 2:** Number of unvaccinated children and total number examined for vaccination status by transit site at the July SIA last day of full activity, Gajraula block, Jyotiba Phule Nagar district, Uttar Pradesh, India, Aug 5^th ^2006.

Site	Unvaccinated	N	%	95%CI
Rail station	174	2,340	7.4	6.5-8.6
Bus stand	186	2,841	6.6	5.7-7.5
Street intersection	143	4,834	3.0	2.5-3.5

## Discussion

In 2006, the number of children vaccinated against polio at areas of mass transit in JP Nagar district increased between the June and July campaigns. The number of vaccinations delivered per shift varied according to team composition and the proportion of immunized children varied by type of transit site.

Transit vaccinations in Amroha Urban and Gajraula (where we implemented a number of changes based on analysis of the June 2006 data) increased between the June and July 2006 SIAs despite an overall decline in the other blocks of the district where we did not implement these changes. An increase in team-shifts contributed to increased number of children vaccinated in Amroha Urban and Gajraula. However, team-shifts also increased by 5.1% in non-intervention blocks and the number of vaccinations declined by 9.5%. Since the pool of traveling children is finite, the addition of team-shifts may not increase vaccinations where contact with these children is already saturated. The number of children in transit also varies seasonally due to holidays, labor migration, etc. The decline in vaccinations in the non-intervention blocks could be related to seasonal changes. This would imply that the increase in vaccinations in the intervention blocks also occurred during a period of fewer children in transit.

In June, teams in Amroha Urban with at least one Muslim vaccinator administered vaccine to almost 13 children more per shift at railway stations (54 vs. 41) than teams without a Muslim vaccinator. However, team composition seemed to have no effect at bus stands or street intersections. In July, all railway teams had at least one Muslim vaccinator, and teams achieved a similar level of performance (vaccinating a median of 57 children per shift). It is important to note other factors for which data were not available could be potential confounders (e.g. the age or experience of the vaccinator, etc). Also, reasons for the site-specific (railway, bus, or intersection) variation of performance in these Amroha Urban teams are unclear. Possible explanations could include a difference in the community composition of travelers at railways and the potentially decreased time a team may have for interaction with families inside train compartments as compared to bus stands or on busy streets. Regardless, the Muslim minority in the area is poorer, has less contact with the health system, and rumor mongers have exploited its fears by alleging that polio vaccination is an effort to sterilize their children [6]. Our data suggest that changing the composition of teams to match that of the local community may increase the number of children vaccinated.

The disparity between intervention and other blocks suggests that the improved program outcome could be a result of the interventions, primarily planning based on previous results with the assignment of appropriate team numbers to different sites (optimization of team assignments), improved training, improved representation of minority groups on teams, and centralized management (operations conducted by a single district level manager instead of multiple block level managers). While we cannot determine which interventions were most effective, each is a plausible contributor to improved performance. For example, having a dedicated trainer for teams may have provided more effective training experiences leading to better performance. More teams or increased shifts at a site may have provided a more rational work load, and facilitated immunization of more children during peak hours. Further clarification of the effectiveness of specific interventions may be the subject of subsequent evaluations.

According to the new monitoring data we collected by recording the vaccination status of children encountered at transit sites, the proportion of unvaccinated children in Gajraula block during the July 2006 SIA was lowest at street intersections. In contrast, at rail and bus centers a higher proportion of unvaccinated children were found. This is likely due to the limited geographic scope of the SIAs. They were not national programs, and thus at rail and bus centers a higher proportion of children might be arriving from distant areas where SIA's were not in progress, leading to a higher proportion of unvaccinated children at these sites compared to street transit sites (where a higher proportion of children were local residents exposed to the SIA).

As expected, the proportion of unvaccinated children at transit sites rapidly declined over the course of the July SIA as more children were vaccinated in house-to-house and transit activities. The proportion of total unvaccinated children in the district is routinely estimated through a street survey conducted by district staff and hired monitors following the end of each SIA. This routine Gajraula block street survey for July 2006 SIA examined 659 children of whom 18 (2.7%) were unvaccinated (unpublished program data). This street survey result of 2.7% was similar to the proportion of unvaccinated children identified in our evaluation by transit team vaccinators at the end of full activity at street intersections (3.0%). Thus, directing transit vaccination teams to record the number of already vaccinated children provides data similar to the independent street survey but with an eight fold greater sample size and without any additional time or cost. Furthermore, recording the number of children checked increased the accountability of transit teams.

As an observational evaluation of immunization operations, our study is subject to several limitations. We did not randomize blocks to intervention and non-intervention groups. We selected Amroha Urban and Gajraula because they were a program priority with the most transit sites and represented 53% of the total transit vaccinations in the district. Similarly, existing teams were evaluated without randomization of personnel or teams, and the associations identified may have been mediated by unidentified confounders. A displacement effect though, where increased attention and resources were devoted to the intervention blocks from the non-intervention blocks, is unlikely as the planning and operations of transit activities in non-intervention blocks was and remained under the care of block level management. Another limitation was the limited duration of follow-up. Changes in performance were measured only during the subsequent July SIA due to resource constraints. The possibility of a Hawthorne effect [7], where some short-term improvements are caused by simply observing worker performance, cannot be ruled out. Each of these factors increases complexity and makes causal inference difficult.

## Conclusions

Operational evaluation projects with analysis of vaccination data can improve the outcome of polio vaccination campaigns. Other studies have evaluated elements of the polio program in India, [8-11] but have not examined vaccination operations at mass transit sites. We conclude that the analysis of program data can guide interventions to both increase the proportion of children reached with vaccine and help monitor vaccination coverage. Some strategies that seek to increase delivery beyond the standard booth vaccination program (where vaccines are offered at fixed sites to which children must travel), such as house-to-house vaccinations, are carefully planned, monitored, and evaluated. Similar care should be extended to transit site activities to further advance polio eradication. We also found that recording the number of already vaccinated children encountered in the context of transit team activities provides useful data with little effort. Similar evaluations of operational data may be helpful in improving immunization delivery in other settings in India or elsewhere.

## Competing interests

The authors declare that they have no competing interests.

## Authors' contributions

NKS, AT, VJ conceived the study. NKS and AT collected the data. NKS, SB, YH, JW analyzed the data and all authors provided critical contributions to the paper. All authors read and approved the final manuscript.

## Pre-publication history

The pre-publication history for this paper can be accessed here:

http://www.biomedcentral.com/1471-2458/10/243/prepub
